# Supplementary material for: Multifunctional flexible membranes from sponge-like porous carbon nanofibers with high conductivity
Source: Nat Commun. 2019 Dec 6;10:5584. doi: 10.1038/s41467-019-13430-9 (PMC6897989; doi:10.1038/s41467-019-13430-9)
Supplement: Supplementary file 1 — Supplementary Information [file 41467_2019_13430_MOESM1_ESM.pdf]

Supplementary Information

Multifunctional Flexible Membranes from Sponge-Like Porous Carbon Nanofibers with High Conductivity

Yan et al.

TABLE OF CONTENTS

Supplementary Methods..4

Supplementary Tables...5

Supplementary Table 1. Specific surface areas and pore volumes of PCNFs that produced with different PVA contents...5

Supplementary Table 2. Comparison of energy and power densities of supercapacitors between our work and the reported work...5

Supplementary Figures..6

Supplementary Figure 1. A sketch of using chemical crosslinking electrospinning to synthesize as-spun fibers...6

Supplementary Figure 2. Viscosity characterizations of PVA particles............................7

Supplementary Figure 3. Digital photos of the white as-spun and the brown oxidized fibrous films..8

Supplementary Figure 4. SEM figures and diameter histograms of the as-spun and oxidized fibers..9

Supplementary Figure 5. FTIR spectra of the as-spun fibers and oxidized fibers...........10

Supplementary Figure 6. TG analysis of PVA, PTFE and the as-spun fibers.................11

Supplementary Figure 7. XRD test of the PCNF-1200 ^{o}C...12

Supplementary Figure 8. XPS characterization of the PCNFs..13

Supplementary Figure 9. SEM images of the PCNFs that produced with different precursors and temperatures...14

Supplementary Figure 10. SEM images of the PCNFs that produced with different PVA contents..15

Supplementary Figure 11. The diameter histograms of PCNFs that produced with different PVA contents..16

Supplementary Figure 12. SEM figures and diameter histograms of different PCNFs that produced with the same PVA contents but different pyrolysis temperatures..................17

Supplementary Figure 13. TG curves of the three different PCNFs that demonstrated in Figure S12...18

Supplementary Figure 14. Field emission SEM images of PCNFs (PVA-50%) with different magnifications..19

Supplementary Figure 15. TEM characterizations of carbon structures in PCNFs.........20

Supplementary Figure 16. Pore distributions of PCNFs that produced with different PVA contents..21

Supplementary Figure 17. Figures of the freestanding PCNF films and the commercial Al foils that used for battery current collectors...22
Supplementary Figure 18. Schematic illustration of matter transfer routes within the tri-modal pore structures of the PCNFs...23
Supplementary Figure 19. The liquid-storage performance of the PCNFs......................24
Supplementary Figure 20. Charge-discharge curves of the supercapacitors at 1, 2, 5, 8 and10 A g^{-1}...25
Supplementary Figure 21. The carbon plates that were used for sandwiching the as-spun NF films for the pyrolysis at high temperatures...26
Supplementary Figure 22. SEM images of the as-spun fibers and the corresponding products after pyrolysis...27
Supplementary Figure 23. Conductivity comparison of the PCNF films that fabricated under different gas atmosphere...28
Supplementary Figure 24. Morphology comparison of the PCNF films that fabricated under different gas atmosphere...29
Supplementary Figure 25. A photo of using the PCNF films as a wire to light a bulb...30
Supplementary References...31

Supplementary Methods

Illustration of the possible reactions between B and N_2.

In this paper, the crosslinked PVA-BA-PTFE would transform into B-doped PCNFs. According to the reaction:

$$2B(s) + N_2(g) = 2BN(s)$$

Where N_2: $\Delta fHm = 0$ Kj mol^{-1}; $\Delta fGm = 0$ Kj mol^{-1}; $Sm = 191.5$ J mol^{-1} K^{-1};

B: $\Delta fHm = 0$ Kj mol^{-1}; $\Delta fGm = 0$ Kj mol^{-1}; $Sm = 5.86$ J mol^{-1} K^{-1};

BN: $\Delta fHm = -254.39$ Kj mol^{-1}; $\Delta fGm = -228.45$ Kj mol^{-1}; $Sm = 14.81$ J mol^{-1} K^{-1};

Therefore, for this reaction,

$\Delta H = -254.39$ Kj mol^{-1}; $\Delta S = -86.8$ J mol^{-1} K^{-1}; $\Delta G = -228.4$ kJ $mol^{-1} < 0$,

According to Gibbs function, the reaction occurs spontaneously when $\Delta G < 0$, which means that B and N_2 can react at room temperature. A high temperature could accelarate the reaction.

Supplementary Tables

Supplementary Table 1. Specific surface areas and pore volumes of PCNFs that produced with different PVA contents.

Samples	S_{BET}（m^2/g)	V_{total}（cm^3/g)	V_{meso}（cm^3/g)
PCNFs-30% PVA	**750.6**	**0.58**	**0.42**
PCNFs-50% PVA	**633.6**	**0.54**	**0.38**
PCNFs-70% PVA	**537.5**	**0.34**	**0.26**

Supplementary Table 2. Comparison of energy and power densities of supercapacitors between our work and the reported work. The tested voltage window was 0~3.5 V.

	Energy Density (Wh/kg)	Power density (KW/kg)
Our work	**42.77**	**1.75**
Ref 1	**4.5**	**9.6**
Ref 2	**7.8**	**0.25**
Ref 3	**42**	**11.2**
Ref 4	**16.67**	**7.0**
Ref 5	**63.7**	**1.0**
Ref 6	**90**	**0.105**
Ref 6	**70**	**0.55**
Ref 6	**55**	**0.105**
Ref 6	**85**	**9.0**
Ref 7	**12**	**3.0**
Ref 8	**35**	**0.75**
Ref 9	**6.0**	**0.052**
Ref 10	**53.5**	**0.16**
Ref 11	**53.1**	**0.98**

Supplementary Figures

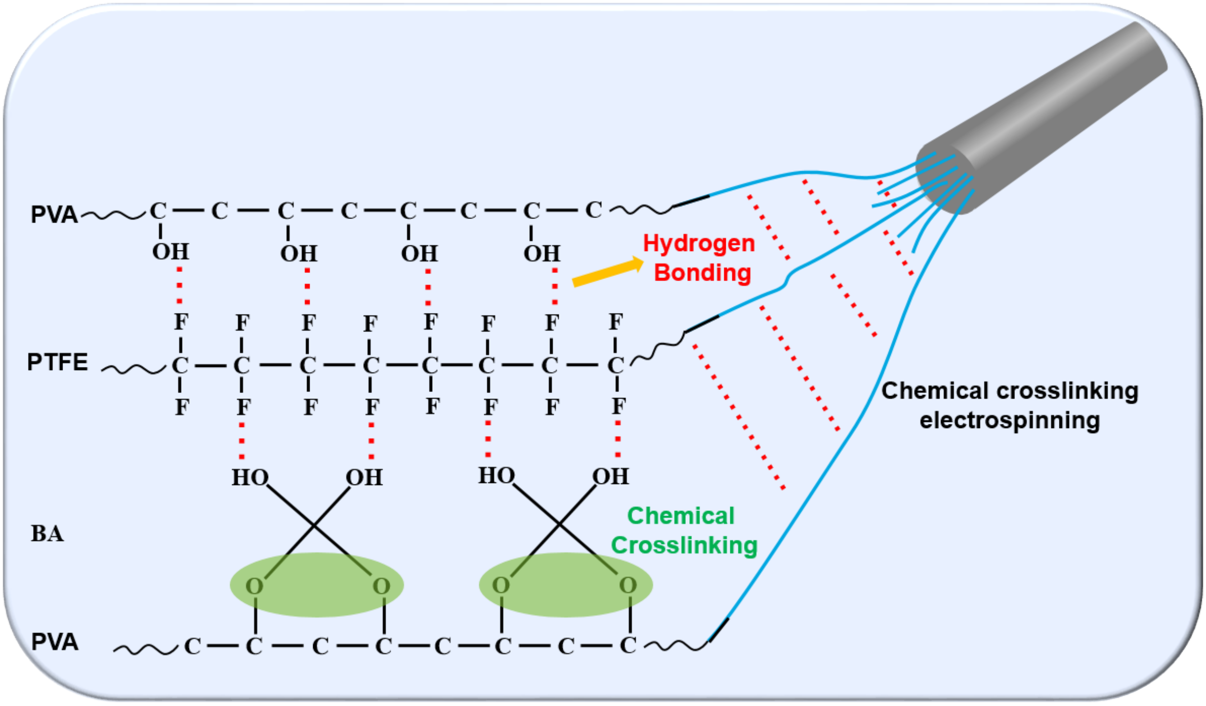

Supplementary Figure 1. A sketch of using chemical crosslinking electrospinning to synthesize as-spun fibers.

In this spinning sol-system, BA connected with PTFE and PVA through hydrogen bonding and chemical bonding, respectively. In addition, there were also hydrogen bonding between PVA and PTFE.

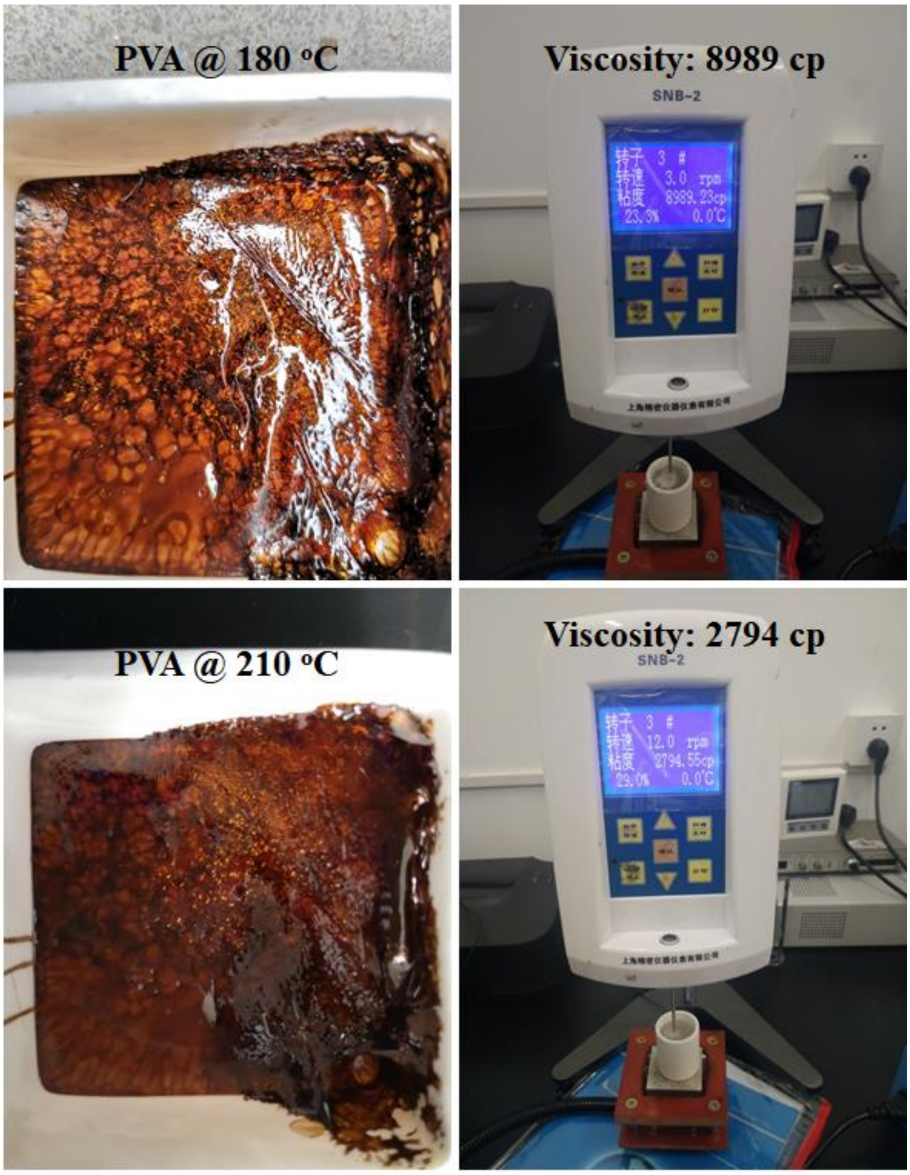

Supplementary Figure 2. Viscosity characterizations of PVA particles.

The tests were taken at a humidity-controlled room and the samples were heated in air. At 180 ^{o}C, the PVA had a higher viscosity of 8989 cp, while at 210 ^{o}C, it exhibited a low viscosity of 2794 cp. Of note, once turning off the heater, the PVA could quickly form a film.

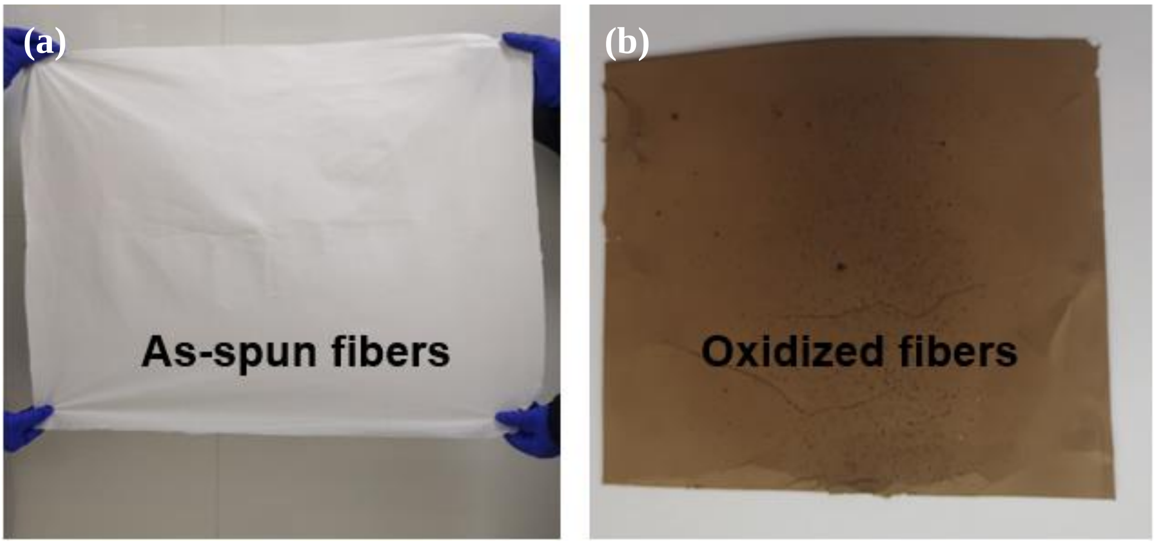

Supplementary Figure 3. Digital photos of the white as-spun and the brown oxidized fibrous films.

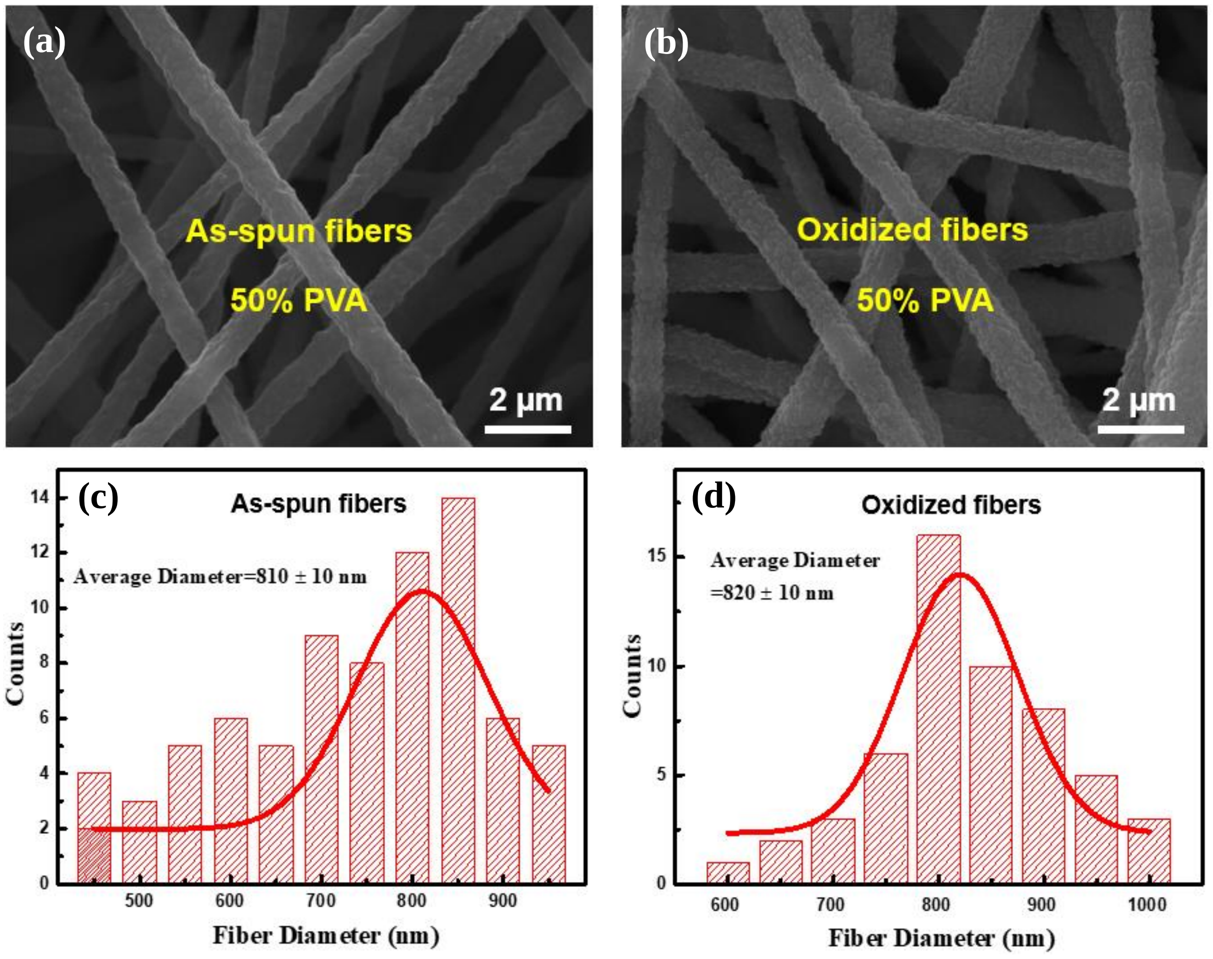

Supplementary Figure 4. **SEM figures and diameter histograms of the as-spun and oxidized fibers.**

The fibers were produced with 50% PVA. The average fiber diameters of the as-spun and oxidized fibers were 810 nm and 820 nm, respectively.

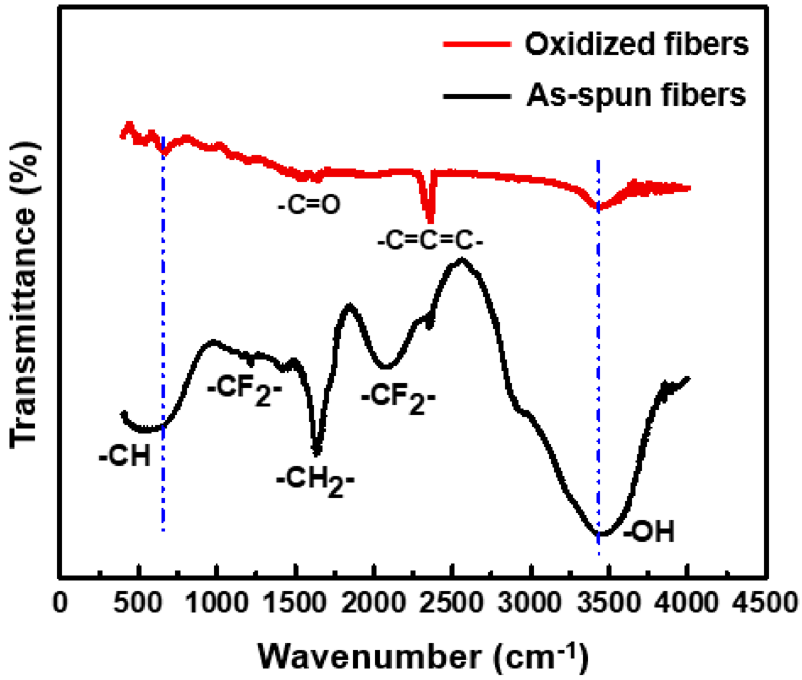

Supplementary Figure 5. FTIR spectra of the as-spun fibers and oxidized fibers.

The as-spun fibers presented a lot of characteristic peaks of C-F bonds. After oxidation, these peaks disappeared. Whereas, there were new characteristic peaks of C=C and C=O bonds. Source data are provided as a Source Data file.

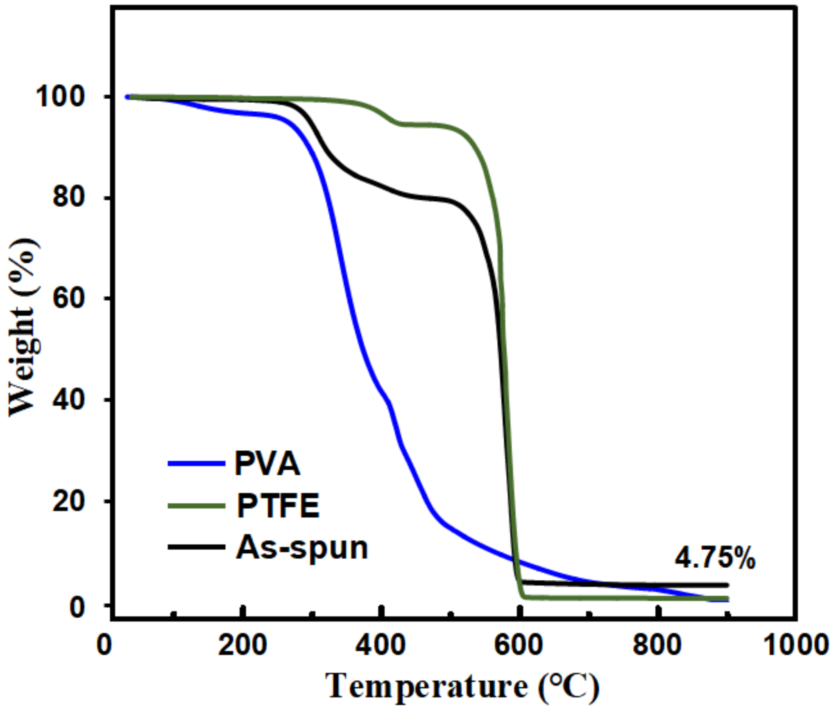

Supplementary Figure 6. **TG analysis of PVA, PTFE and the as-spun fibers.**

The tests were conducted in N_2-atmosphere at a heating rate of 5 ℃ min^{-1}. The weight of PVA decreased continuously along with the temperature. However, both the PTFE and the as-spun fibers became stable after 600 °C, indicating that the final carbon was from both the PVA and PTFE. The total carbon yield was 4.75% according to the TGA. Source data are provided as a Source Data file.

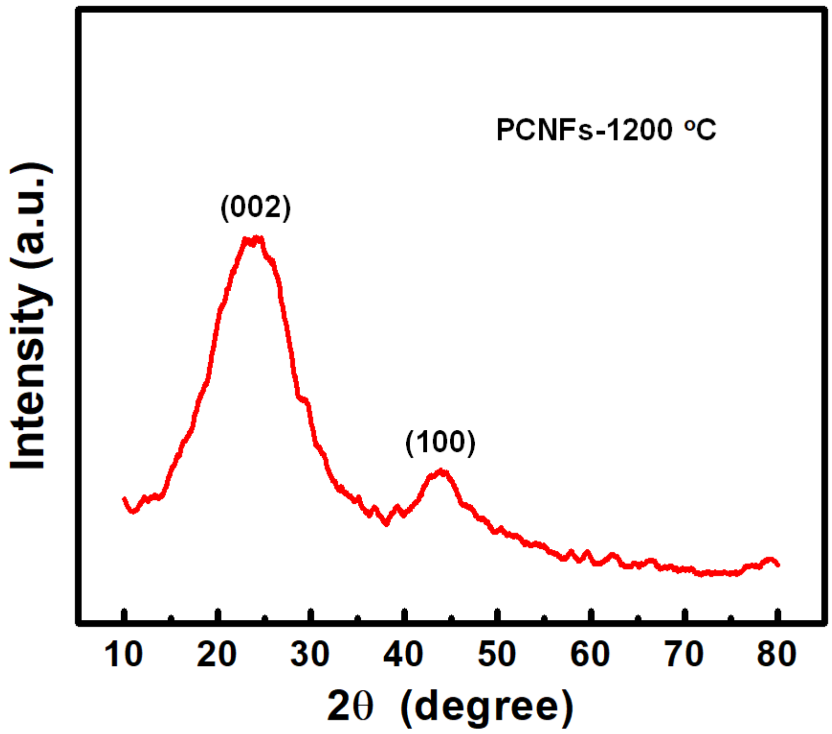

Supplementary Figure 7. XRD test of the PCNF-1200 °C.

The spectra indicated a high graphitization degree of carbon in the PCNFs. Source data are provided as a Source Data file.

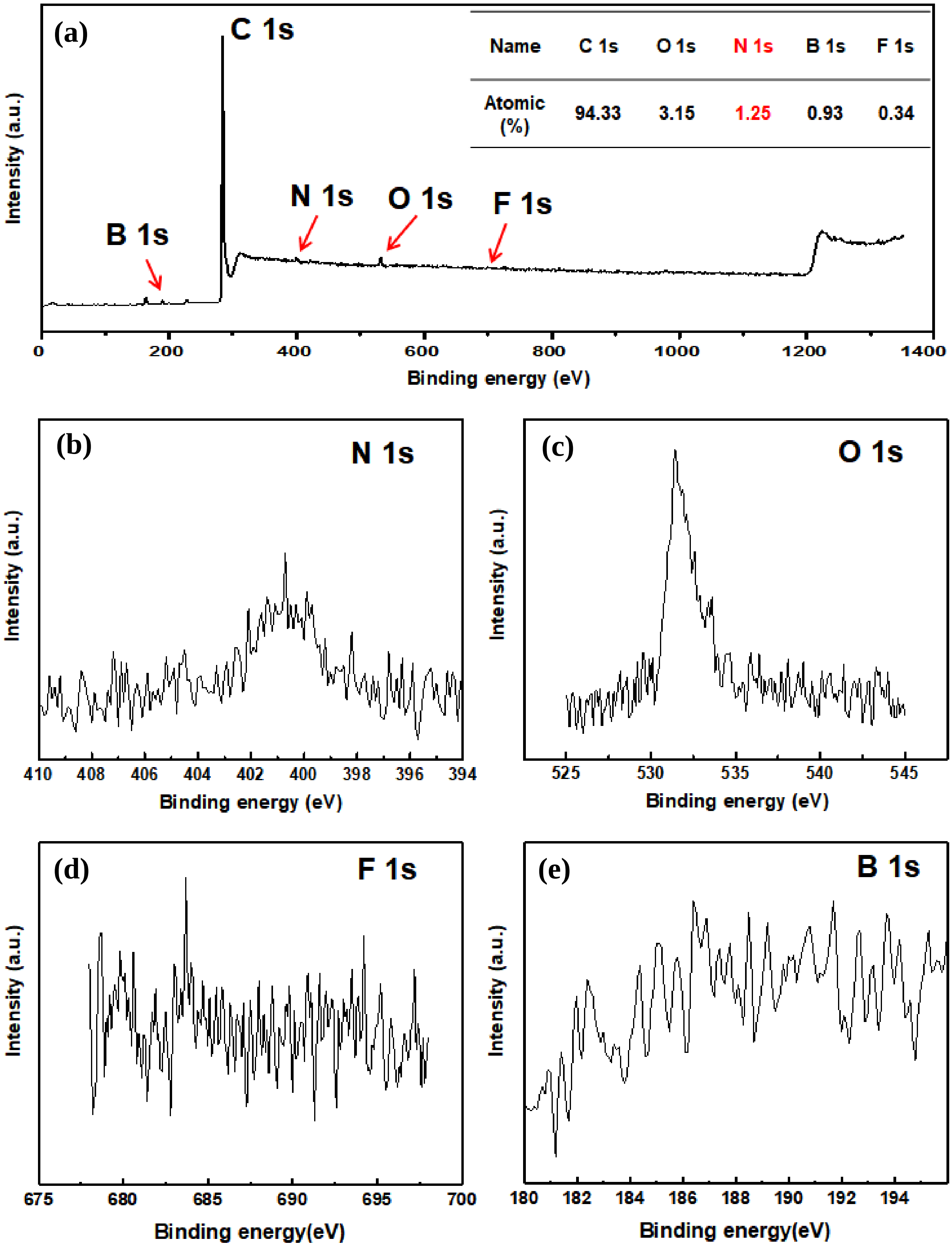

Supplementary Figure 8. XPS characterization of the PCNFs.

The atomic percents of C, O, N, B and F were 94.33%, 3.15%, 1.25%, 0.93% and 0.34%, respectively. (**b**-**e**) demonstrate the spectra of N 1s, O 1s, F 1s and B 1s, respectively. Source data are provided as a Source Data file.

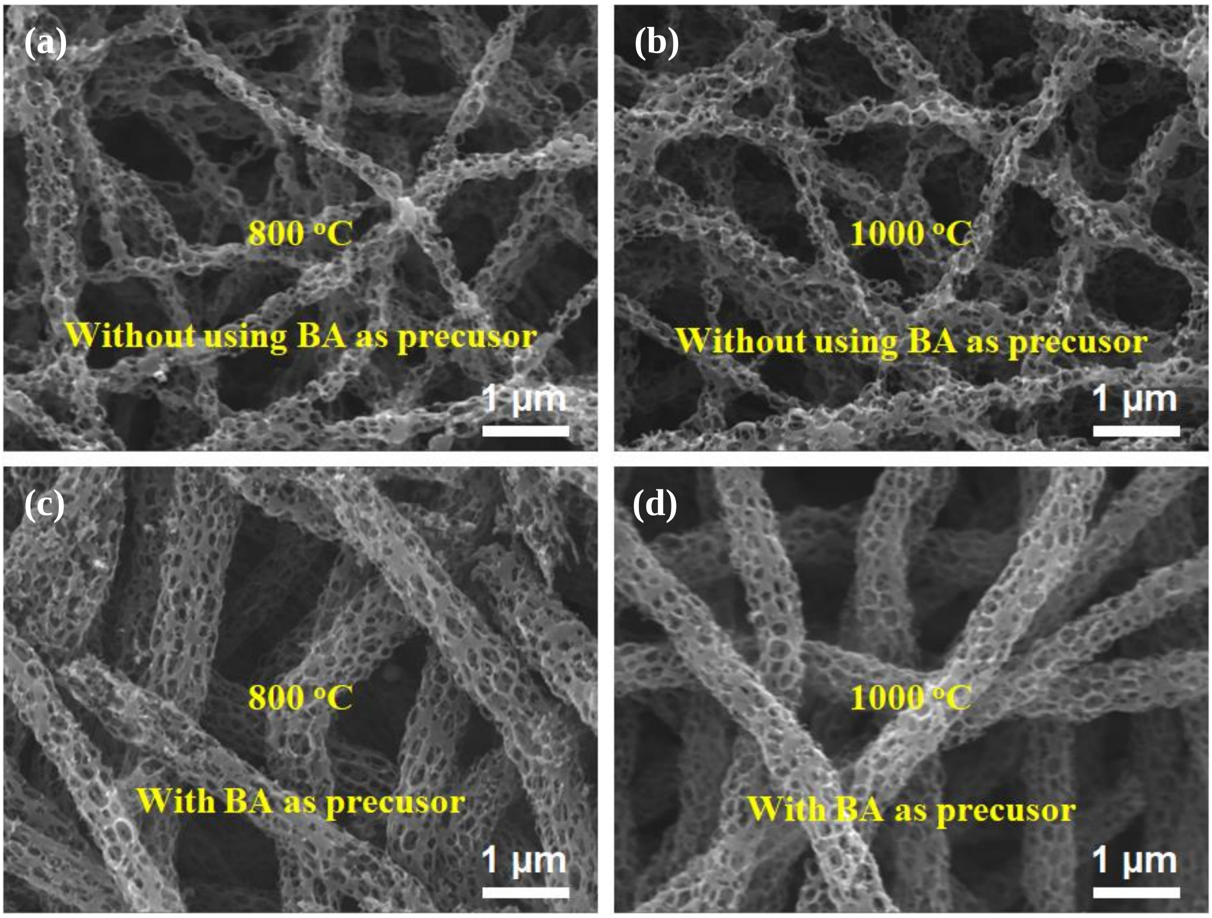

Supplementary Figure 9. **SEM images of the PCNFs that produced with different precursors and temperatures.**

(**a**, **b**) Without using and (**c**, **d**) with BA as one of the precursors at two different pyrolysis temperatures of 800 ^{o}C and 1000 ^{o}C. Without using BA, the PCNFs demonstrated curved and thinner morphology compared with the PCNFs that produced with BA as the precursor, indicating the crosslinking effect of using BA in the sol system.

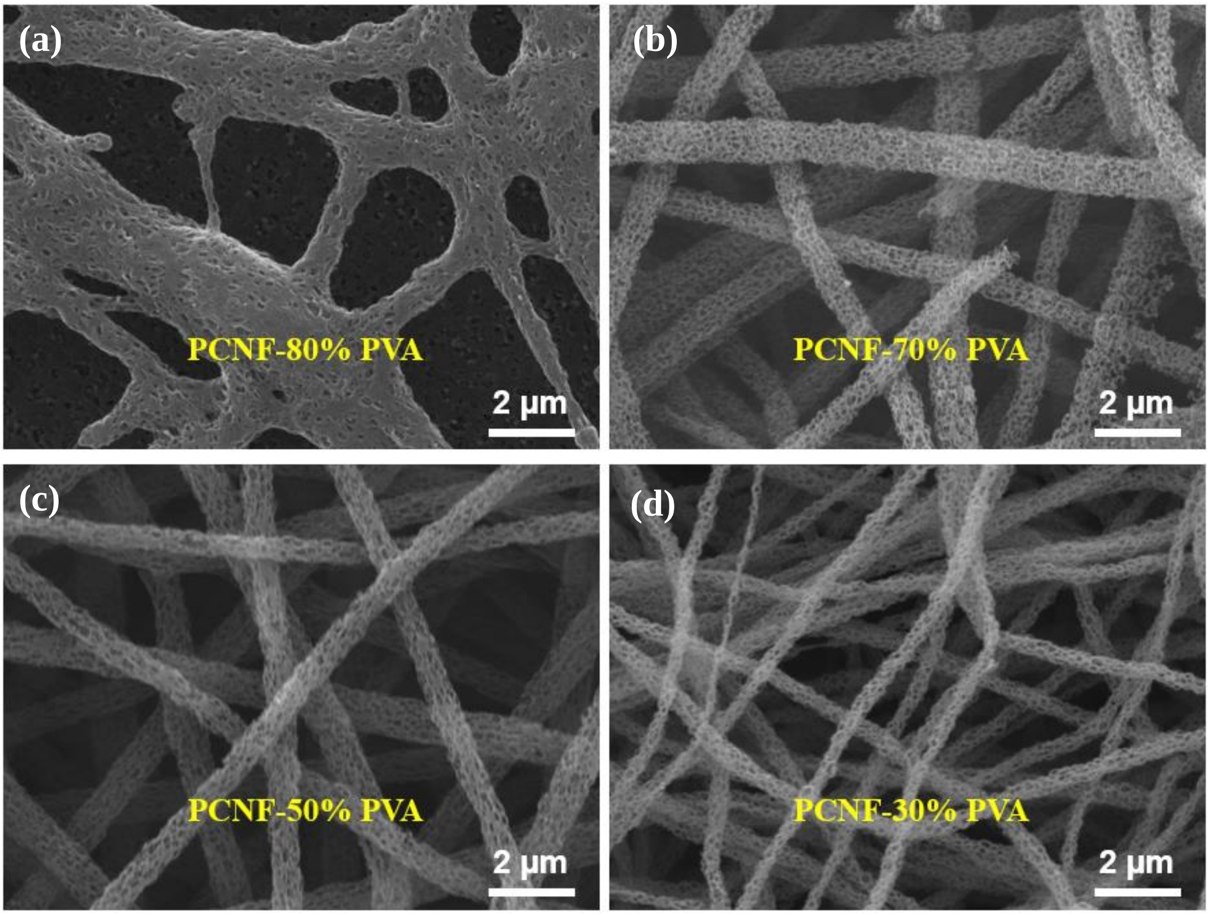

Supplementary Figure 10. **SEM images of the PCNFs that produced with different PVA contents.**

(**a**) 80 wt.%, (**b**) 70 wt.%, (**c**) 50 wt.% and (**d**) 30 wt.%. The diameters of the PCNFs decreased with the decreasing of the PVA contents, but all of the PCNFs had sponge-like porous structures.

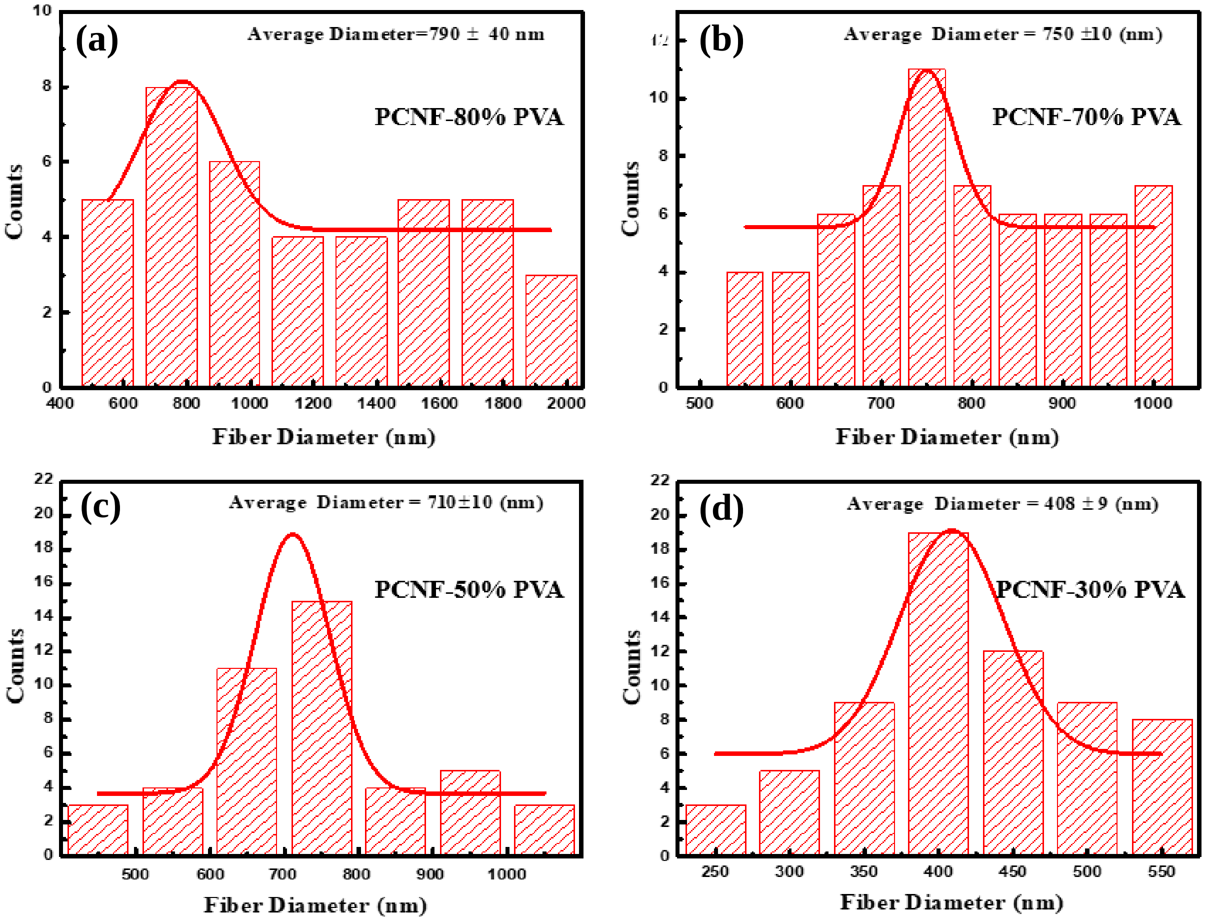

Supplementary Figure 11. **The diameter histograms of PCNFs that produced with different PVA contents**.

The average fiber diameters of the four kinds of PCNFs were 790 nm, 750 nm, 710 nm and 408 nm, respectively.

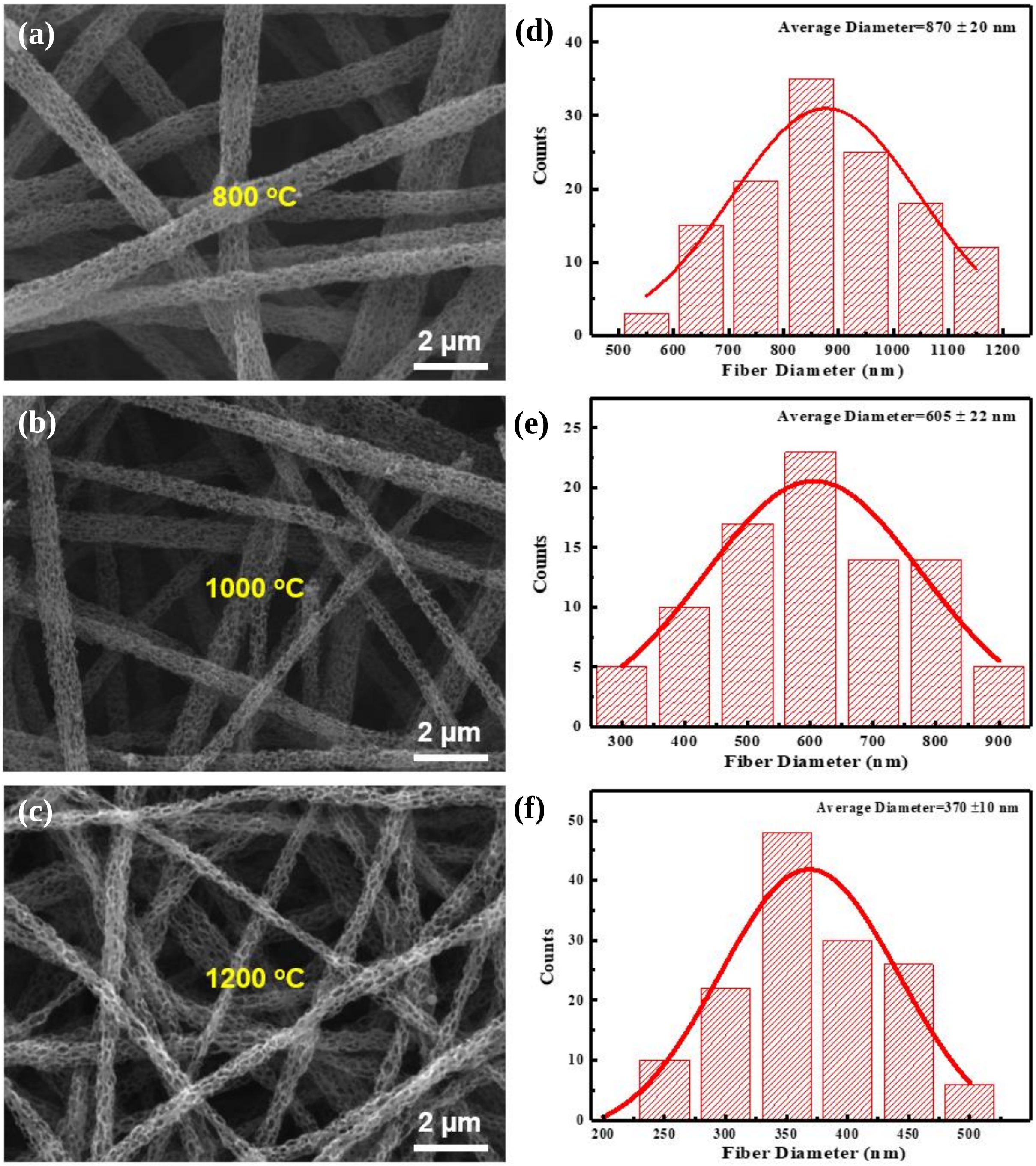

Supplementary Figure 12. SEM figures and diameter histograms of different PCNFs that produced with the same PVA contents but different pyrolysis temperatures.

The PVA content was 30%. The average fiber diameters of the PCNF-800 ^{o}C, PCNF-1000 ^{o}C and PCNF-1200 ^{o}C were 870 nm, 605 nm and 370 nm, respectively.

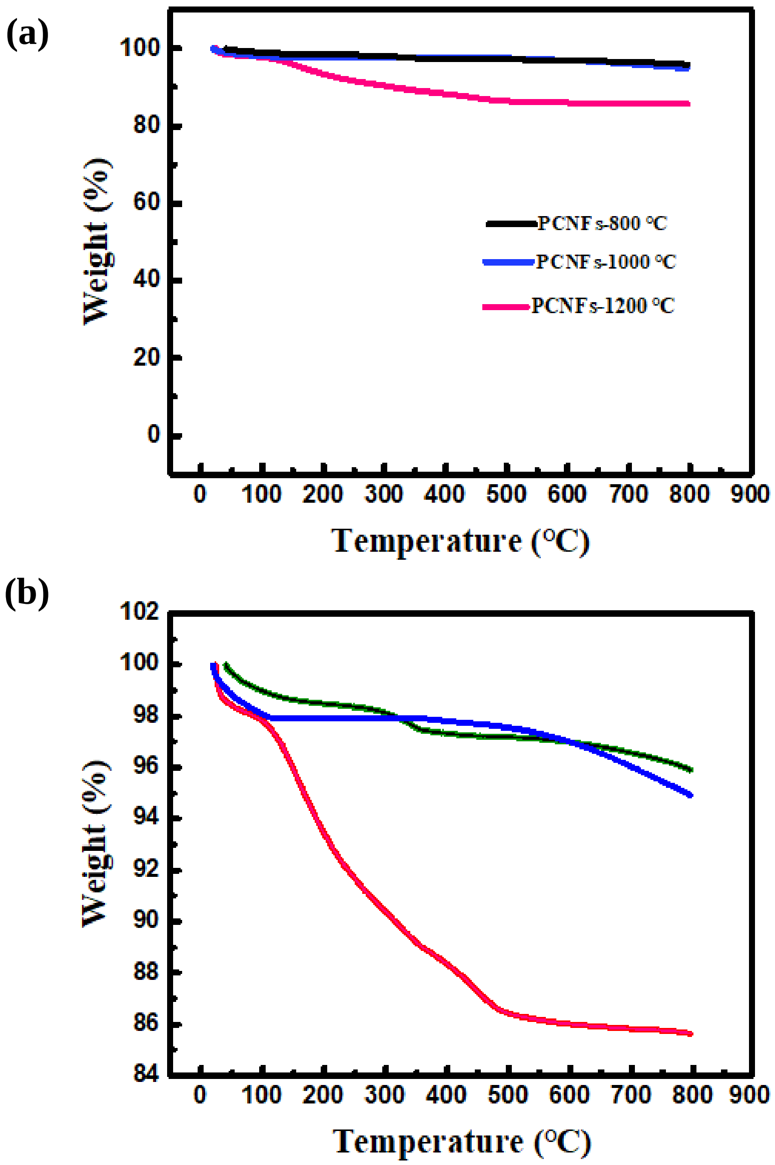

Supplementary Figure 13. TG curves of the three different PCNFs that demonstrated in Figure S12.

The TG analysis was tested in N_2-atmosphere at a heating rate of 5 ℃ min^{-1}. Source data are provided as a Source Data file.

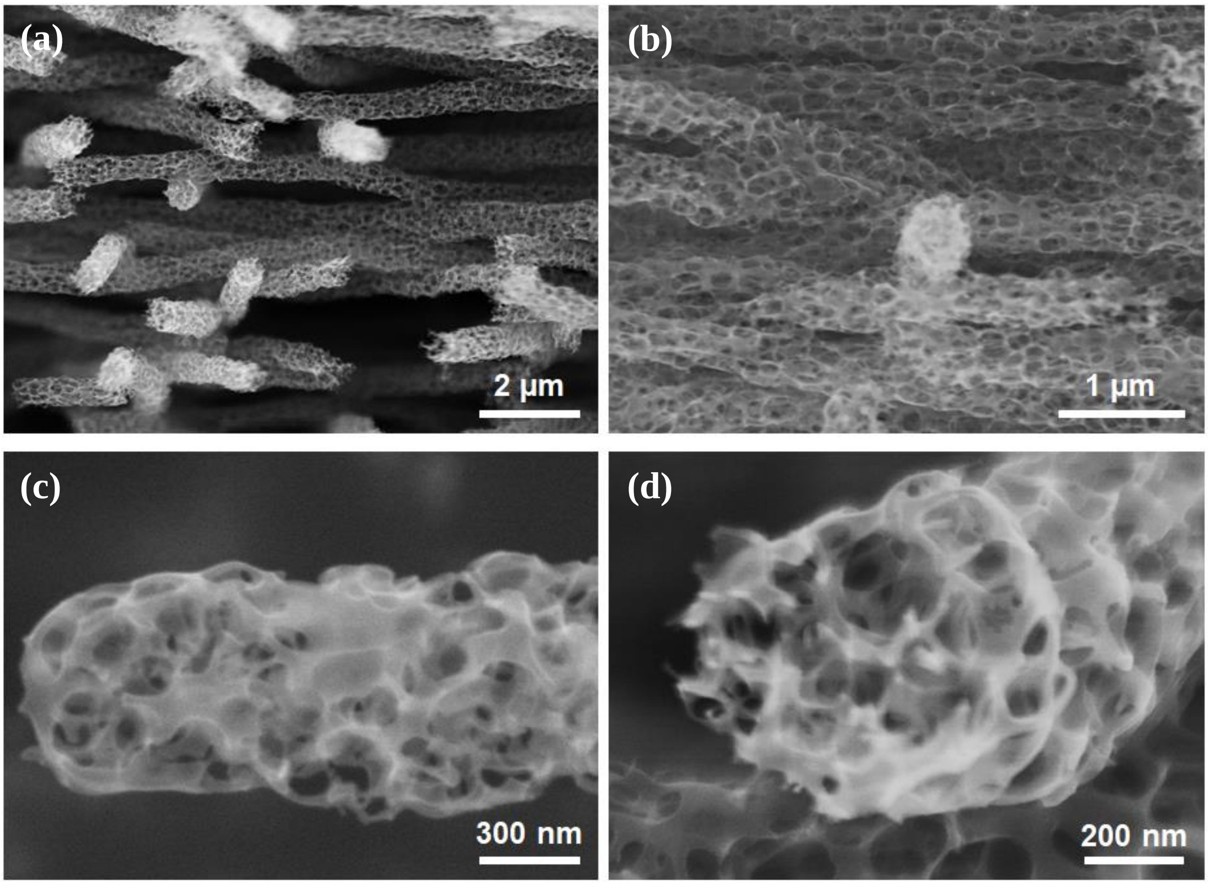

Supplementary Figure 14. Field emission SEM images of PCNFs (PVA-50%) with different magnifications.

The PCNFs contained continuous macro pores throughout the full fibers.

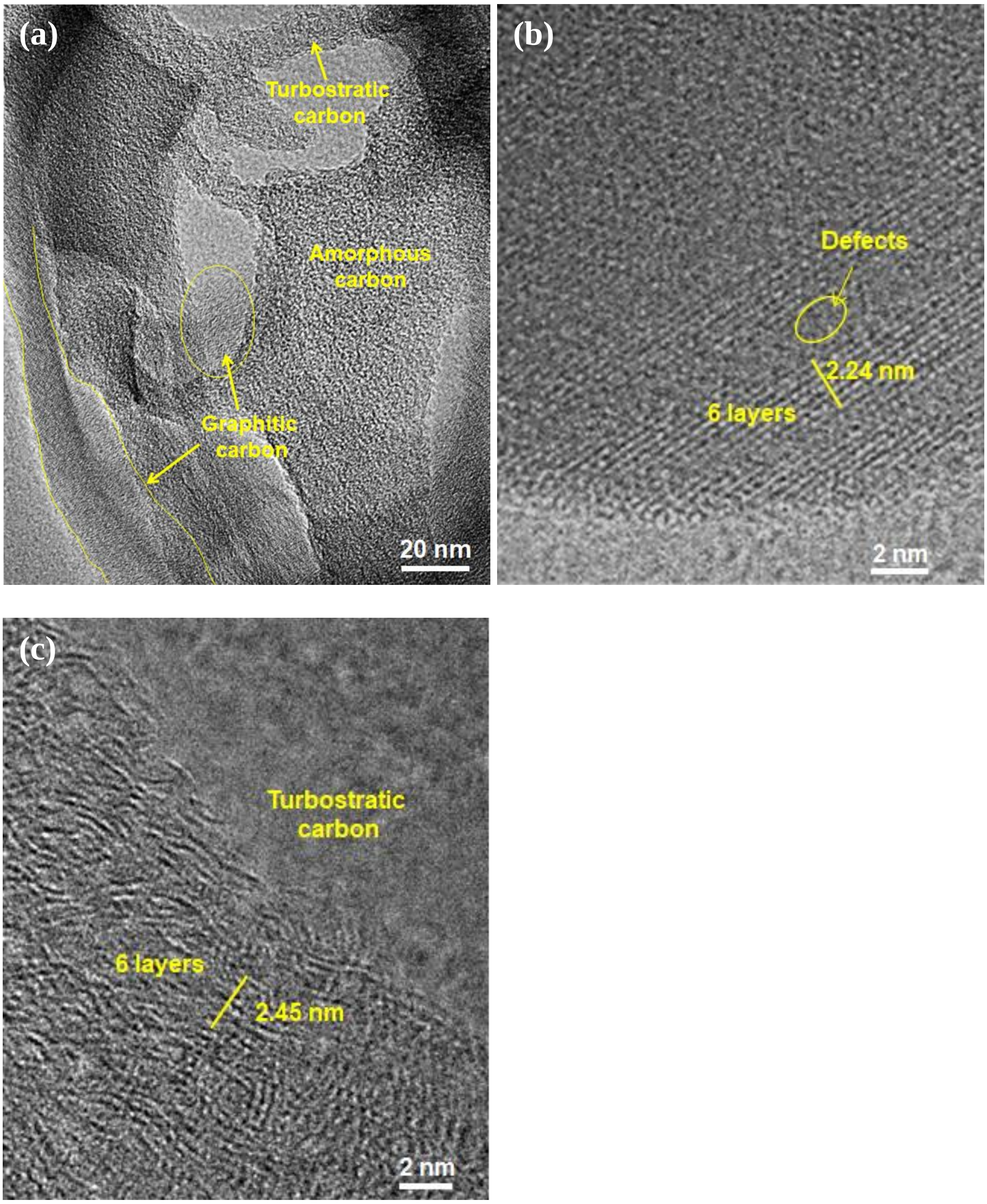

Supplementary Figure 15. TEM characterizations of carbon structures in PCNFs.

There were obviously layered carbon structures with an average inter-planar distance of ~0.41nm.

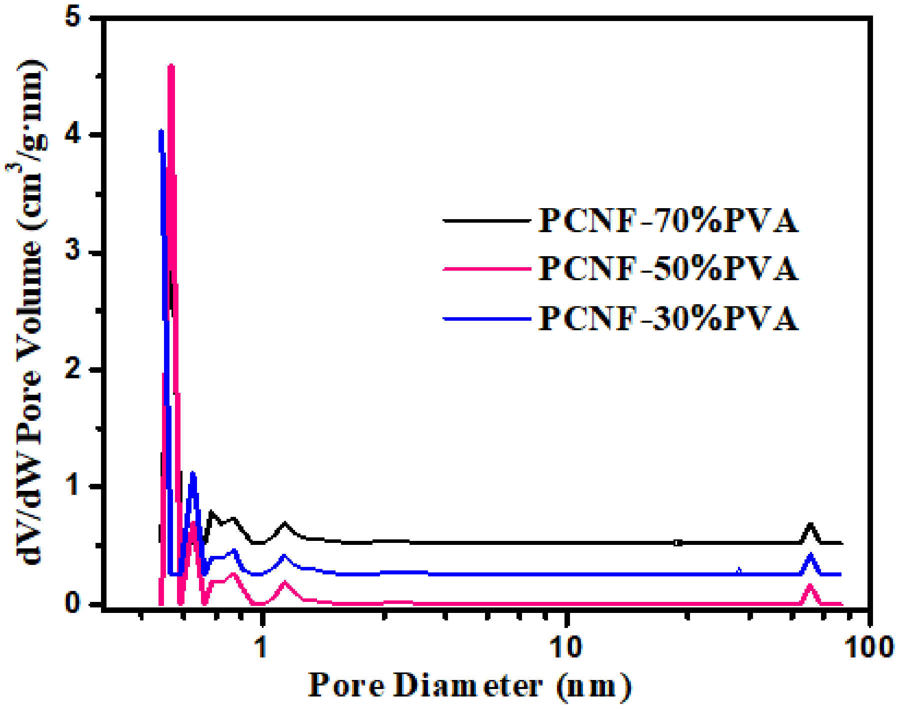

Supplementary Figure 16. **Pore distributions of PCNFs that produced with different PVA contents.**

These three samples demonstrated the same characteristic peaks, indicating they had the similar macro pore-size, meso pore-size and micro pore-size, although their pore volumes were different. Source data are provided as a Source Data file.

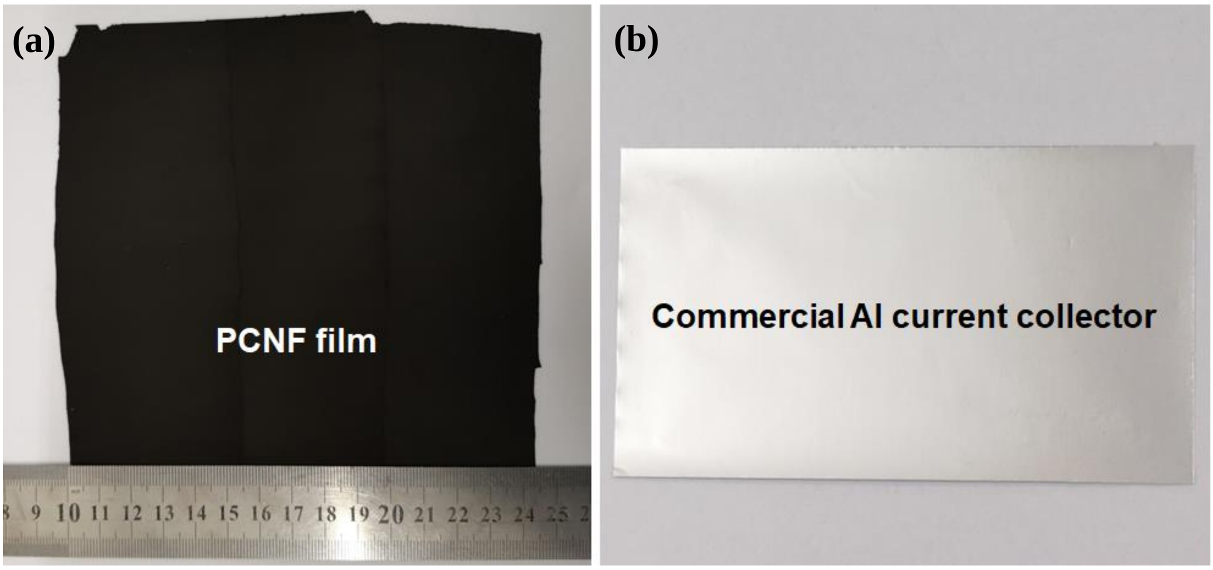

Supplementary Figure 17. Figures of the freestanding PCNF films and the commercial Al foils that used for battery current collectors.

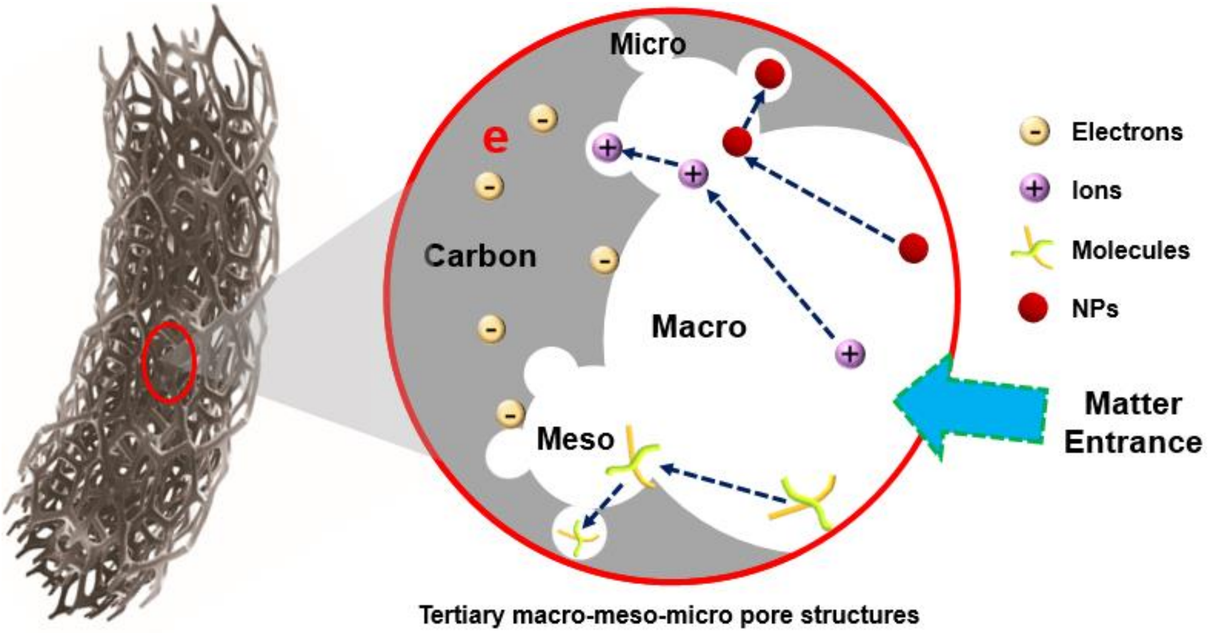

Supplementary Figure 18. Schematic illustration of matter transfer routes within the tri-modal pore structures of the PCNFs.

As shown in the diagram, a large amount of electrons, ions, molecules and particles could rapidly access into the macro pores, and then transferred into the inner spaces of the meso and micro pores.

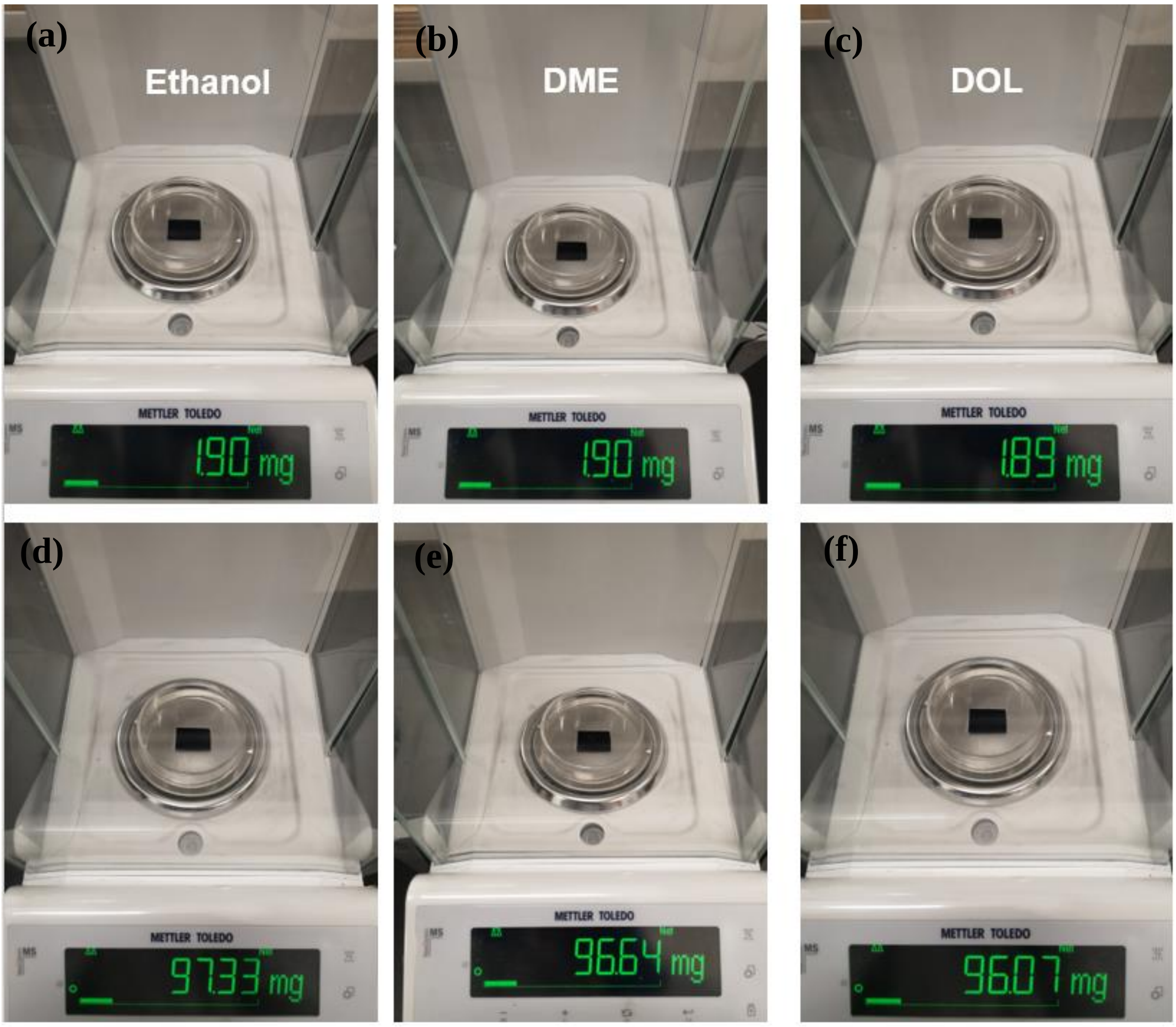

Supplementary Figure 19. The liquid-storage performance of the PCNFs.

(**a, d**) The weights of the PCNFs before and after absorbing ethanol. (**b, e**) The weights of the PCNFs before and after absorbing DME. (**c, f**) The weights of the PCNFs before and after absorbing DOL. The storing mass ratios for all these three liquids were ~50.

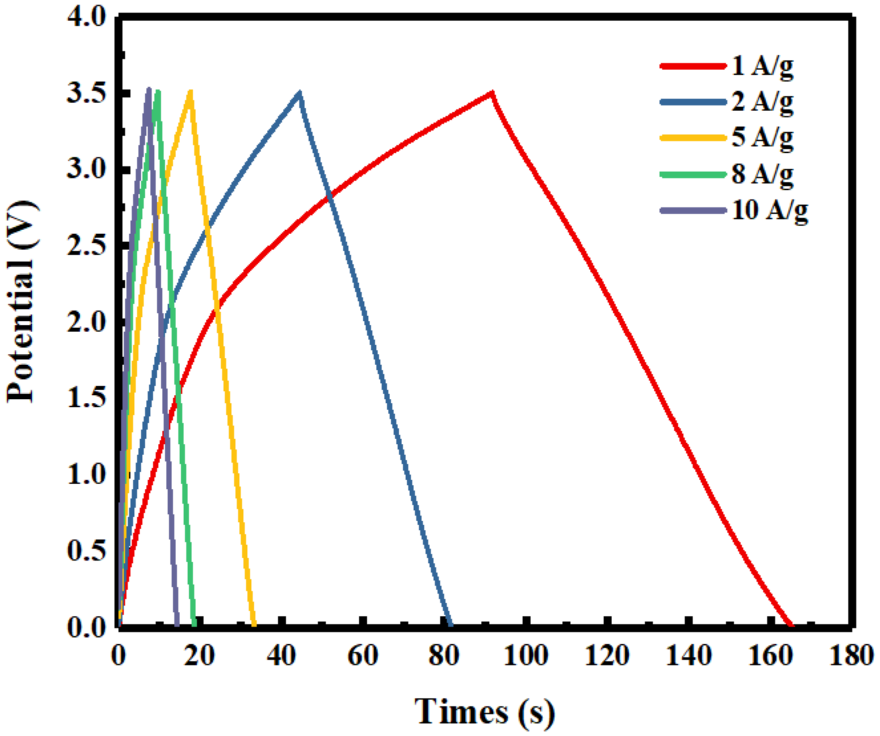

Supplementary Figure 20. Charge-discharge curves of the supercapacitors at 1, 2, 5, 8 and10 A g^{-1}. Source data are provided as a Source Data file.

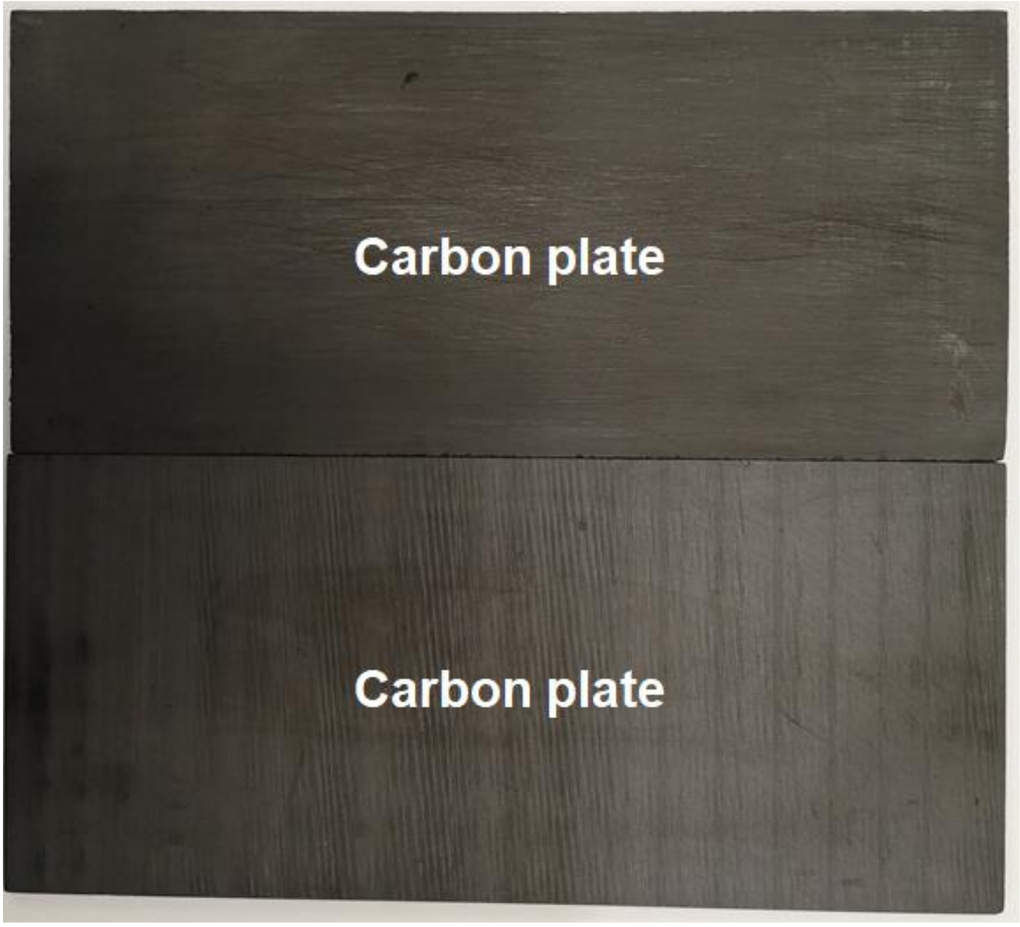

Supplementary Figure 21. The carbon plates that were used for sandwiching the as-spun NF films for the pyrolysis at high temperatures.

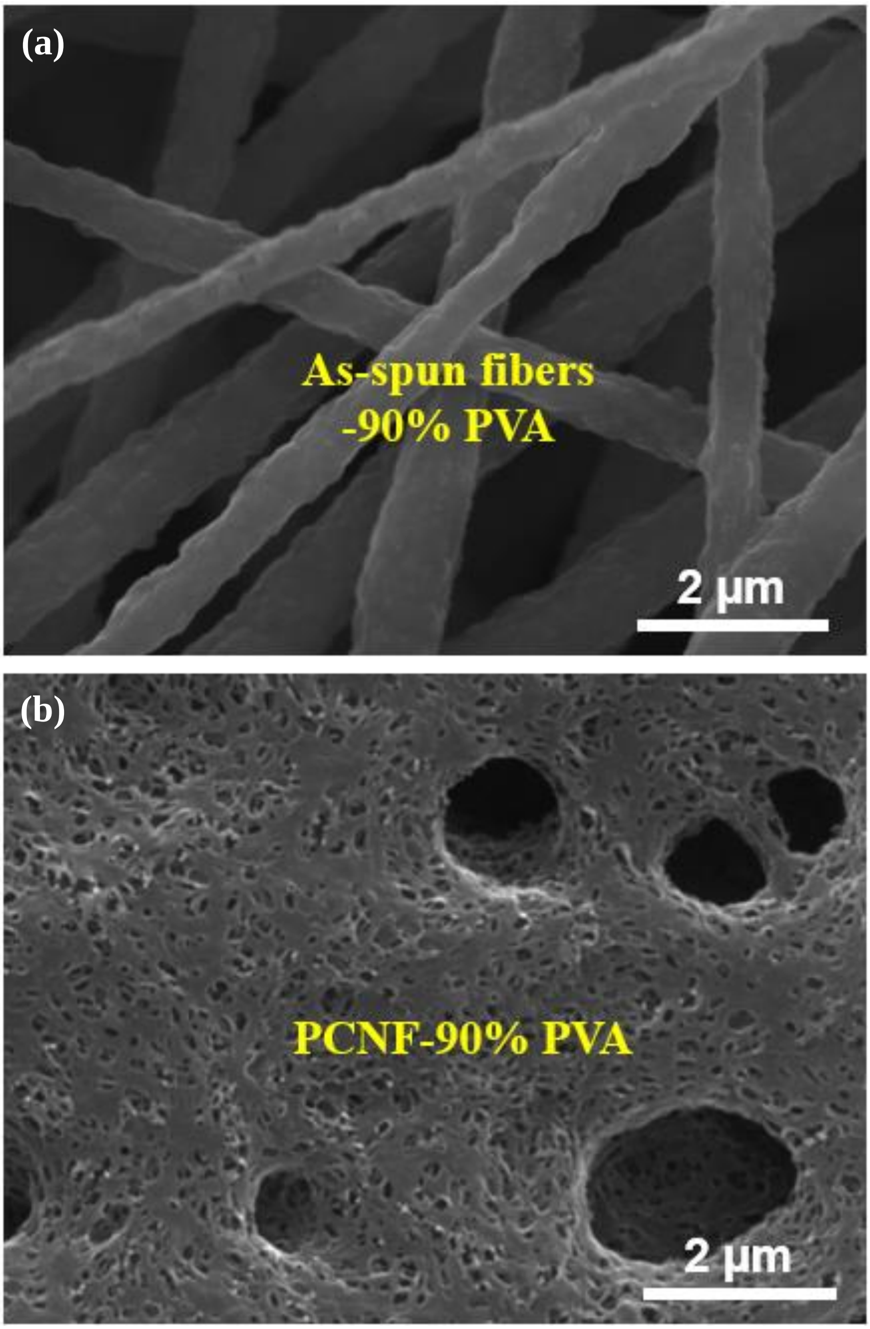

Supplementary Figure 22. SEM images of the as-spun fibers and the corresponding products after pyrolysis.

The PVA content was 90 wt.% in the PVA-PTFE composites, which could be electrospun into fibers. However, after oxidation and pyrolysis, the PCNFs would no longer be formed due to the diffusion of the PVA during the high temperature pyrolysis.

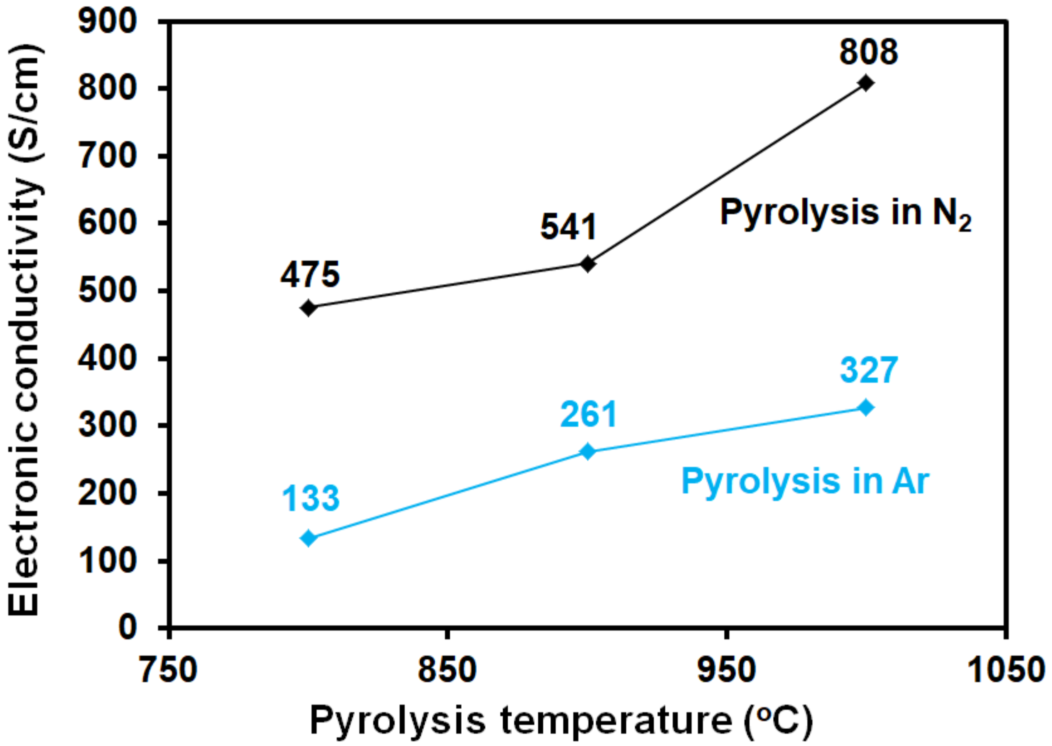

Supplementary Figure 23. Conductivity comparison of the PCNF films that fabricated under different gas atmosphere.

At three different pyrolysis temperatures of 800, 900 and 1000 °C, the PCNFs that created under Ar always exhibited lower conductivity than the PCNFs that fabricated under N_2.

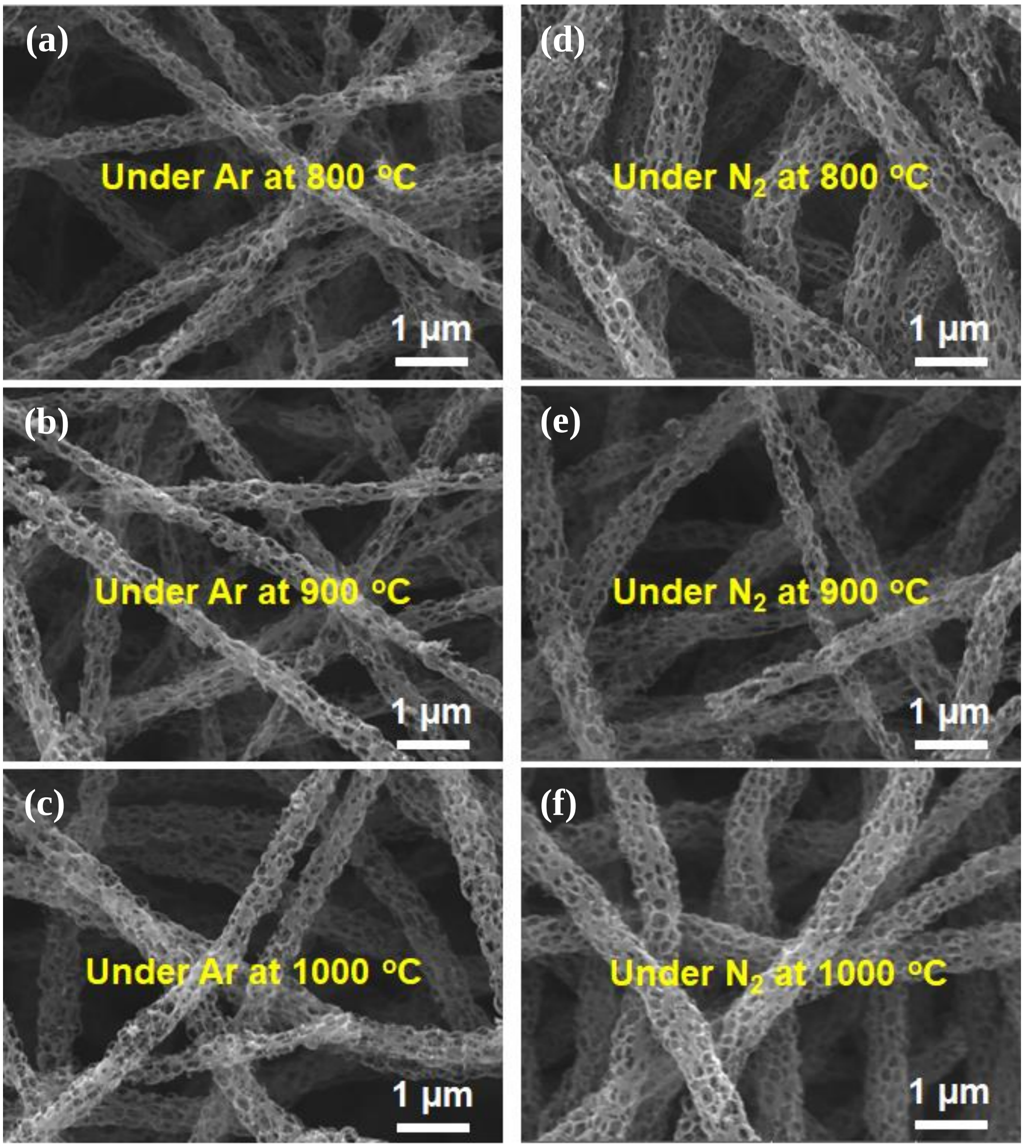

Supplementary Figure 24. Morphology comparison of the PCNF films that fabricated under different gas atmosphere.

At three different pyrolysis temperatures of 800, 900 and 1000 ^{o}C, the PCNFs had similar morphology.

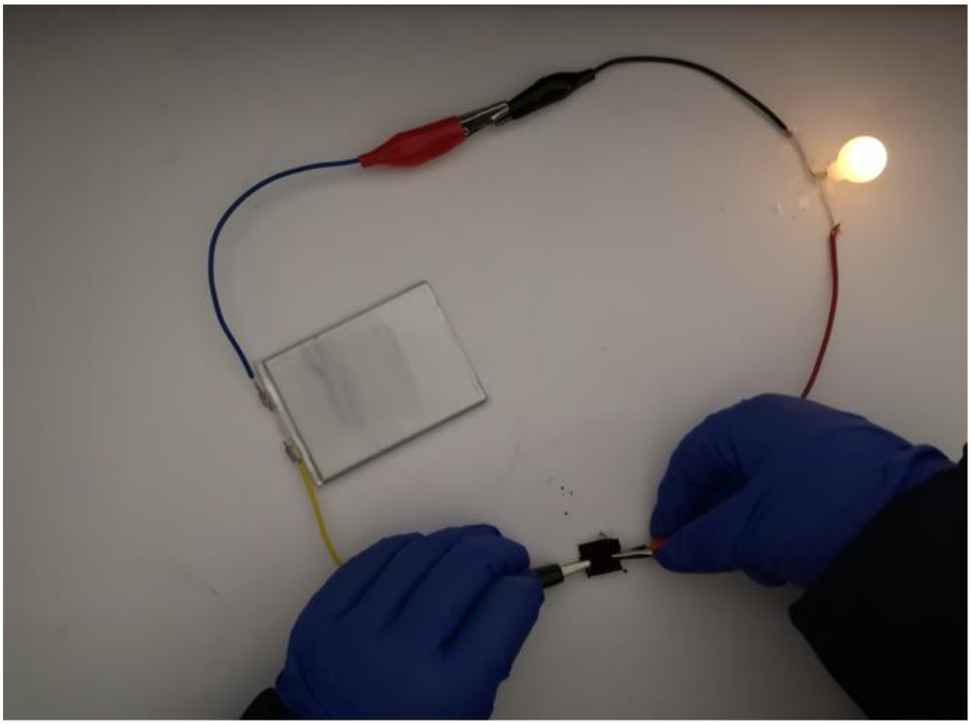

Supplementary Figure 25. A photo of using the PCNF films as a wire to light a bulb.

Supplementary References

1. Zhou, Z. et al. Block copolymer-based porous carbon fibers. *Sci. Adv.* **5**, (2019).
2. Huang, Y. et al. Biobased nano porous active carbon fibers for high-performance supercapacitors. *ACS Appl. Mater. interfaces* **8**, 15205-15215 (2016).
3. Lee, W. S. V. et al. Low Li^{+} insertion barrier carbon for high energy efficient lithium-ion capacitor. *ACS Appl. Mater. interfaces* **10**, 1690-1700 (2018).
4. Wang, S., Dryfe, R. A. W. Graphene oxide-assisted deposition of carbon nanotubes on carbon cloth as advanced binder-free electrodes for flexible supercapacitors. *J. Mater. Chem. A* **1**, 5279-5283 (2013).
5. You, B., Jiang, J., Fan, S. Three-dimensional hierarchically porous all-carbon foams for supercapacitor. *ACS Appl. Mater. interfaces* **6**, 15302-15308 (2014).
6. Weng, Z. et al. Controlled electrochemical charge injection to maximize the energy density of supercapacitors. *Angew. Chem. Inter. Edit.* **52**, 3722-3725 (2013).
7. Aboutalebi, S. H. et al. High-performance multifunctional graphene yarns: toward wearable all-carbon energy storage textiles. *ACS nano* **8**, 2456-2466 (2014).
8. Wang, H. et al. High performance supercapacitor electrode materials from electrospun carbon nanofibers in situ activated by high decomposition temperature polymer. *ACS Appl. Energy Mater.* **1**, 431-439 (2018).
9. Liang, T. et al. Popcorn-derived porous carbon for energy storage and CO_2 capture. *Langmuir* **32**, 8042-8049 (2016).
10. Qin, F. et al. Asphaltene-based porous carbon nanosheet as electrode for supercapacitor. *ACS Sust. Chem. Eng.* **6**, 15708-15719 (2018).
11. Liu, C. et al. Graphene-based supercapacitor with an ultrahigh energy density. *Nano Lett.* **10**, 4863-4868 (2010).
